# Mechanical Properties and Microstructural Characterization of Laser Welded S32520 Duplex Stainless Steel

**DOI:** 10.3390/ma14195532

**Published:** 2021-09-24

**Authors:** Hany S. Abdo, Asiful H. Seikh

**Affiliations:** Mechanical Engineering Department, College of Engineering, King Saud University, P.O. Box 800, Al-Riyadh 11421, Saudi Arabia; habdo@ksu.edu.sa

**Keywords:** laser butt welding, weld strength, microstructure, hardness

## Abstract

This paper investigates an experimental design of laser butt welding of S32520 duplex stainless steel, which has been passed out with the help of a pulsed Nd: YAG laser supply. The intention of the present research is to learn the impact of beam diameter, welding speed, and laser power on the superiority of the butt weld. The individuality of butt joints has been characterized in terms of tensile properties, fractography, and hardness. It was noticed that unbalanced particle orientations indirectly produce a comparatively fragile quality in the laser welded joint. The outcome of varying process parameters and interaction effect of process parameters on ultimate tensile strength and micro hardness were studied through analysis of experimental data. With different process parameters, the heat energy delivered to the material was changed, which was reflected in tensile strength measurement for different welded samples. From this present research, it was shown that, up to a certain level, an increase in process parameters amplified the tensile strength, but after that, certain level tensile strength decreased with the increase in process parameters. When process parameters exceeded that certain level, the required amount of heat energy was not delivered to the material, resulting in low bead width and less penetration, thus producing less strength in the welded joint. Less strength leads to more ductile weld joints. Microhardness was higher in the weld zone than in the base region of welded samples. However, the heat affected zone had a high microhardness range.

## 1. Introduction

It is evident that laser welding machineries are broadly used in manufacturing industries and automotive industries. As per the property, duplex stainless steel (DSS) characterizes a category of stainless steels by means of a dual microstructure made of an approximately equivalent magnitude of ferrite and austenite portion. Numerous experimental investigations have been performed to learn the laser welding method. Yang et al. [1] inspected the impact of heat contributions on microstructure along with corrosion manners on heat affected zone for 2205 duplex stainless steel. They established that the reformed austenite content improved with the coarsening of grain boundary austenite and the expansion of intragranular austenite and Widmanstatten austenite, consequently improving the toughness and disturbing corrosion status. Mirshekari et al. [2] offered a comparative investigation on laser welding of NiTi wire with the same and with AISI 304 austenitic stainless steel. It was shown that tensile strength and ductility reduces considerably while NiTi is coupled with AISI 304 due to the arrangement of brittle intermetallic compounds at the weld region for the duration of laser welding. Additionally, they suggest that an appropriate adjustment procedure is essential to upgrade the joint characteristics. Abdo et al. [3] defined the consequences of used inputs on weld bead geometry, which reveals bead width, bead length, and depth of penetration in laser spot welding of AISI 304. Jia et al. [4] described a well-known analysis on dissimilar joints of DP600 and DP980, through fiber laser using focused and defocused beam. Akbari et al. [5] offered a mathematical and experimental analysis of laser welding of titanium alloy and calculated the temperature allocation and heat affected zone. They established, using a lower welding speed, a penetration depth improved for steady pulse duration, pulse frequency, and power. Akman et al. [6], during investigational study on laser welding of thick titanium alloy, established that the proportion of pulse energy to pulse duration is the main significant constraint to classify the penetration depth of the welded joint. Kashaev et al. [7] studied the arc welding method for butt joints and T- joints of Ti-6Al-4V via an alloy compatible filler wire. Additionally, they have carried out investigations such as weld morphology, microstructure, and mechanical properties to obtain weld joints through standard form, exclusive of noticeable cracks, pores, and geometrical deficiencies. The final conclusions illustrated that both butt and T-joints contain elevated strength as well as ductility compared to their parent metal. Lei et al. [8] characterized the mechanical and microstructural properties for laser welded samples of Ti-22Al-27Nb alloys. The results revealed that the joint potency at room temperature is comparatively unchanged with the parent material, and the ductile property achieved at weld joints was 56% of the parent material. This research also showed that when temperature increases the strength and ductility of a weld, the properties of our samples did not match those of the parent material. Shanmgarajan et al. [9] described the laser welding process of 6 mm thick P92 material (Cr-Mo-W-V-Nb steel) considering mechanical properties at room as well as elevated temperatures and metallurgical properties following post-weld heat treatment at 760 °C for 3 h. This research explained that superior quality of fusion through deep penetration devoid of several weld imperfections and no coarse-grained heat affected zone was produced. Furthermore, superior microhardness was found on the fusion and heat affected zone compared to the parent material. Hosseini et al. [10] estimated the authority of heat input in several welding sequences based on the microstructure of the heat affected zone for TIG welding of 2507 super duplex stainless steel. Zhang et al. [11] explored gas tungsten arc welding and fluxed cored arc welding and characterized the microstructure, impact toughness, and pitting corrosion resistance of duplex stainless-steel welded joints by means of dissimilar shielding gas configurations. This study revealed that impact toughness and pitting resistance will be elevated if N_2_-supplemented shielding gas is used for welding. Asif et al. [12] investigated the effects of heat effort upon the mechanical properties such as joint strength, toughness, microhardness, and metallurgical and corrosion characterization of UNS S31803 duplex stainless steel for solid-state continuous drive friction welding. In this research, it was noticed that there is no intermetallic phase within the welded materials, and that toughness was reduced with a superior heat contribution at room temperature. Microhardness and corrosion resistance was greater than before with the enhance of heat input. Capello et al. [13] deliberated the improvement of laser weldability of a category of 22Cr–5Ni–3Mo (UNS S32205) duplex steel. It was established in this study that if we can select an optimized laser parameter in favor of the post-weld surface treatment, we can achieve a superior structural control on the weld bead microstructure. Batahgy et al. [14] examined the outcomes of laser input constraints upon the dimensions and microstructure of fusion zone and mechanical and corrosion properties of duplex stainless steel. In this paper, to achieve welded joints through a satisfactory profile with mechanical and corrosion properties, we optimized the values of laser power and welding speed, defocusing the distance and type of shielding gas. Some numerical simulations were also done for laser welding of duplex steel. Frewin and Scott [15] offered a three-dimensional finite element representation of heat flow throughout pulsed laser beam welding. The result recommended that temperature profile and weld dimensions are important attributes for absorptivity and energy allocation of the laser beam. De et al. [16] presented a two-dimensional axisymmetric finite element analysis of heat flow for the duration of laser spot welding, considering the temperature dependence of physical properties and latent heat of transformations. It was recommended that with their examined technique, one can estimate the accurate weld pool dimensions. Anawa and Olabi [17], using Taguchi method, optimized the welding pool of dissimilar laser welded components. They indicated that the produced model could calculate the fusion zone and shape acceptably. Belhadj et al. [18] made a three-dimensional finite element model to simulate a thermal history of magnesium-based alloys during laser beam welding. They also carried out experimental investigations to confirm the outcome of numerical simulations, which were in good agreement with the experimental results. Abhilash and Sathiya [19] studied the impacts of laser power, welding speed, and focal point position on bead geometry. It was described in this paper that FEM can be a means to calculate bead geometry with a smaller heat input for laser welding. Kumar [20] prepared a three-dimensional finite element model using COMSOL MULTIPHYSICS for 2 mm thick AISI 316L stainless steel sheets through a pulsed laser beam. The maximum/minimum temperature on AISI 316L stainless steel sheets during laser welding was predicted in this study. Ghosh et al. [21] developed a three-dimensional FEM numerical model with non-stationary heat input to examine the laser butt welding method for 2205 duplex stainless steel, considering phase change, to discover the impact of laser power, scanning speed, and beam diameter on thermal properties and construction of weld bead geometry. Sivagurumanikandan et al. [22] studied the impact of input parameters such as welding speed, laser power, focal position, and pulse frequency on strength of laser welded super duplex stainless steel and found the optimum input parameter with the help of response surface methodology. Prabakaran et al. [23] investigated CO_2_ laser beam welding of dissimilar metals, specifically austenitic stainless steel (AISI316) and low carbon steel (AISI1018), using Taguchi-based gray relational analysis, while laser power, welding speed, and focal distance were measured as the input parameter. Ghosh et al. [24] investigated the experimental laser welding process for 2205 duplex stainless steel to verify the impacts of scanning speeds upon the butt weld quality in terms of tensile strength, micro structure, and micro hardness while considering other parameters such as power, beam diameter, and pulse width as invariable. Khalid and Katayama [25] described Fiber laser welding through elevated melting competence, various keyhole approaches, and power density properties that can degrade the heat and melt flow of the molten pool at some stage in welding. This research is meant to investigate the weldability of fiber laser for 5 mm thick AISI 304 austenitic stainless steels. It was evident that laser power, welding speed, and defocusing distance had an immense impact over the bead geometry and weld zone profile but showed no noticeable impact on microstructure and mechanical properties of welds. Lisiecka and Lisiecki [26] explored the authority of fundamental parameters of laser welding, such as laser beam power, welding speed, and energy input, for butt welded stainless steel AISI 304 sheets on behalf of weld shape and joint quality. Abdo et al. [27] researched on pulsed laser welding procedure on dissimilar materials to investigate the mechanical performance, in terms of strength and micro hardness, and microstructural configuration for welded materials. Landowski [28] investigated the microstructure of laser beam welded stainless steel considering a range of welding parameters. In this study, a Ytterbium fiber laser was used to obtain welded samples by not including the filler material for 2205 duplex stainless steel plates. Through this research, it was explained that laser welding parameters impact weld geometry, and there is a connection between laser beam focus position and weld penetration depth. Hosseini et al. [29] studied laser welding of Ti6Al4Valloy to 304 stainless steel with a 1 mm-thick Cu interlayer by varying the laser power and found that the joint strength and the fracture occurrence location is based on laser power ranges.

In this present research, the mechanical properties, in terms of tensile test, fractography, and micro hardness, of laser welded duplex stainless steel were studied by varying laser process parameters in different ranges.

## 2. Materials and Methods

**Figure 1:**
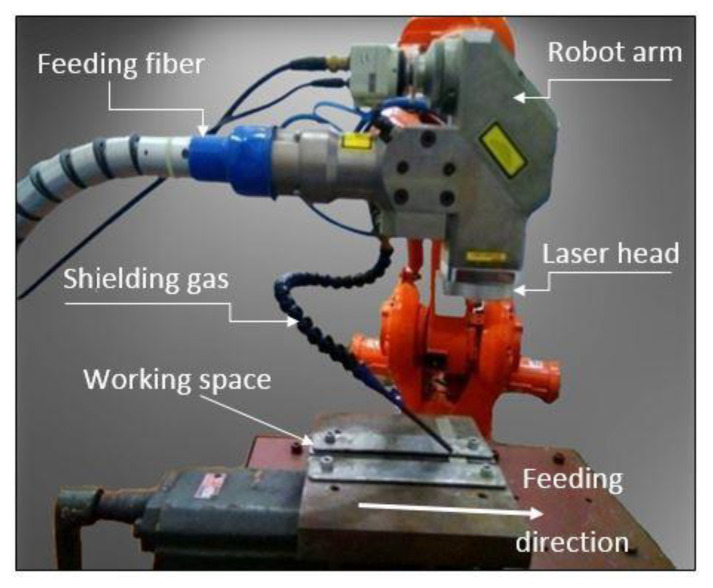
Experimental set-up for laser welding.

Experimental processes are done by a pulsed Nd: YAG laser machine. The experimental arrangement is shown in Figure 1 [24]. The process parameters and sample sizes used are scheduled in Table 1.

**Figure 2:**
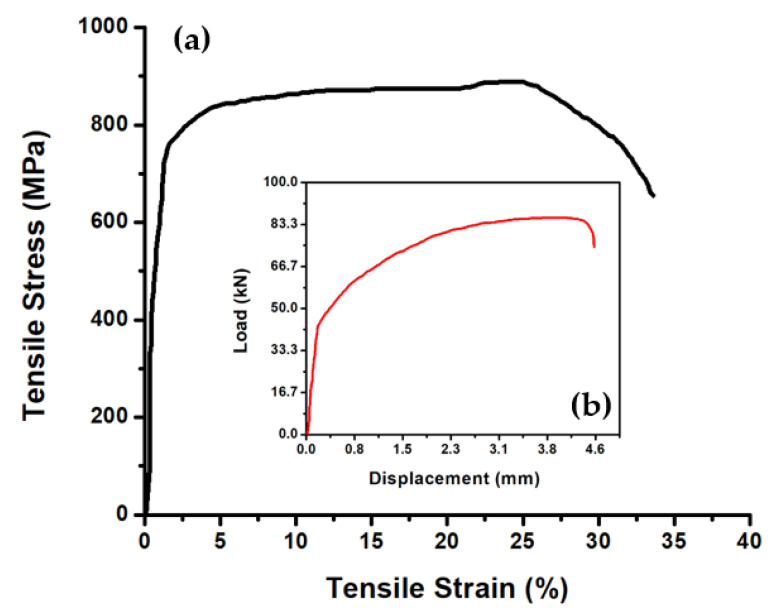
Stress–strain curve (**a**) and deformation curve (**b**) for the base material.

Tensile tests were performed for each base material along with every welded sample, using INSTRON-8801 (Force rating = ±100 kN, Weight = 39 kG, Maximum Pressure = 21 MPa) with a strain rate of 10^−3^ S^−1^. The tensile test samples were arranged as stated by ASTME8 standard. Deformations of the sample were measured using the built-in strain gauge load cell in INSTRON-8801 machine with an accuracy of ±0.5% of indicated load or ±0.005% of load cell capacity (1–100%), whichever is greater. Mechanical characterizations, in numeric values, of each base material are stated in Table 2, and the stress–strain curve is shown in Figure 2.

## 3. Results and Discussions

### 3.1. Tensile Strength Test

**Figure 3:**
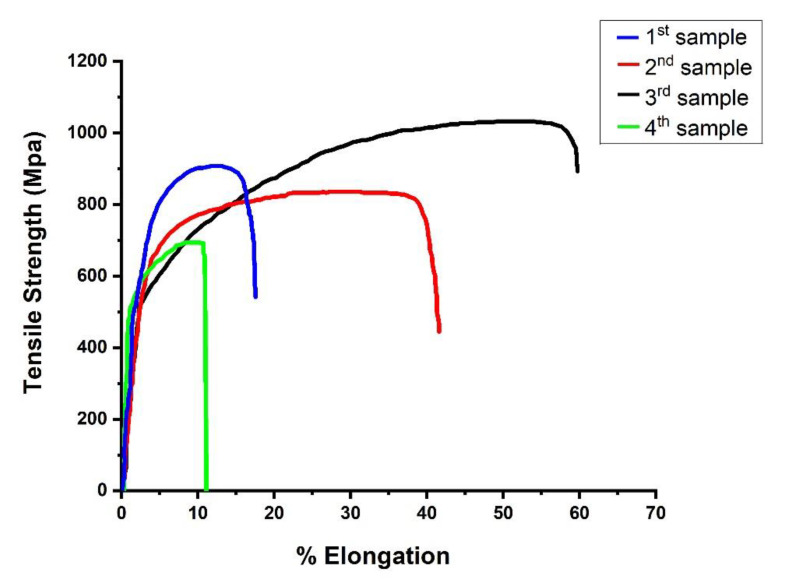
Stress–strain curve for welded samples.

The consequences of welding speed, beam diameter, and laser power upon tensile potency of welded samples are calculated and listed in Table 3. It is observed that ultimate tensile strength (UTS), elongation, and yield strength vary with different process parameters. The fracture position is found in weld region for every case. Figure 3 demonstrates the tensile stress–strain curves of the welded specimens. For Experiment 1 (Blue), the process parameters were: power =500 W, welding speed = 5 mm/s, beam diameter = 0.5 mm. From the Figure, we can see that, for this case, the UTS is 898 MPa, but elongation is significantly less at 19%, and yield strength is 767 MPa. For Experiment 2 (Red), and process parameters were: power = 550 W, welding speed = 6 mm/s, beam diameter = 0.6 mm. From the Figure, we can see that, for this case, the UTS is 811 MPa, but elongation is higher than in previous case at 43%, and yield strength is 660 MPa. For Experiment 3 (Black), and process parameters were: power = 600 W, welding speed = 7 mm/s, beam diameter = 0.7 mm. From the Figure, we can see that, for this case, the UTS is 1007 MPa, but elongation is higher than in the previous case at 62%, and yield strength is 603 MPa, which is lower than in previous case. For Experiment 4 (Green), the process parameters were: power = 650 W, welding speed = 8 mm/s, beam diameter = 1 mm. From the same Figure 3, we can see that, for this case, the UTS is 692 MPa, elongation is 9%, which is very low compared to other cases, and yield strength is 520 MPa. So, for Experiment 3, we can have an optimum process parameter because only in this case did we achieve the highest UTS, and the elongation though yield strength is low. We can conclude that, with the parameters used in Experiment 3, the correct amount of heat input is delivered to the sample, and because of that, it gives the highest strength. When power increases, heat intake by the work piece increases, resulting in more heat penetration, which enhances the chances of deep penetration and wider bead width, resulting in more strength in joints. If the power range exceeds the maximum requirement, then the material can be evaporated due to very high heat, resulting in poor joints that can be easily broken. With an increase in welding speed, the interaction time of the laser beam with the work piece decreases, leading to less heat penetration into work piece, which can be a reason for low strength in joints. Again, if the welding speed is very low, then the interaction time between the laser beam and the work piece increases, which also produces a large amount of heat in the joints due to the same observed material degradation, thus resulting in fragile and brittle joints. As the power density per area decreases with increases in beam diameter, less heat will penetrate the work piece, which leads to less penetration and minor bead width, producing lower strength joints. If the beam diameter decreases, the density of power over the area of joints will increase, thus more heat penetration will occur in joints, which can produce more strength in joints for welding. However, a very small beam diameter will exceed the required amount of heat, which can degrade the joint quality in terms of strength. So, we need to identify the process parameters that can balance the heat input so that only the required, not more and not less, amount of heat can be delivered to the work piece, to obtain high strength as well as more elongation in weld joints. For the process parameters that were used for Experiment 3, the perfect amount of heat was delivered in comparison to the other experimental studies in this paper.

### 3.2. Fractography

**Figure 4:**
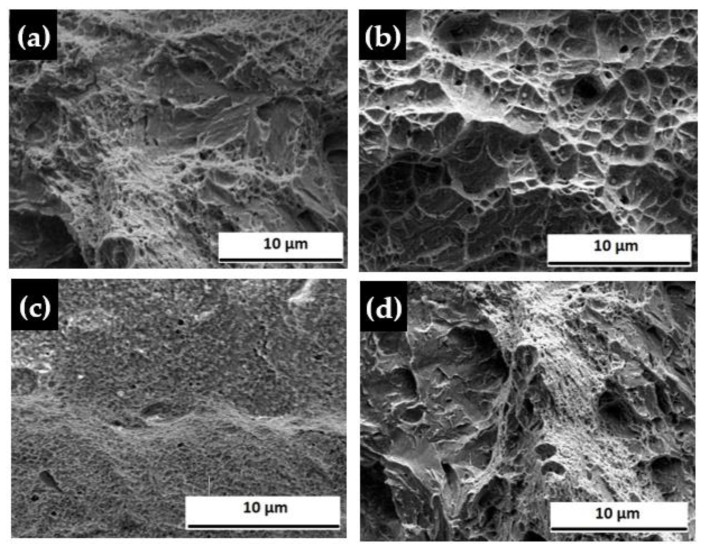
Fracture surface morphology for laser welded samples at different powers: (**a**) 500 W, (**b**) 550 W, (**c**) 600 W, and (**d**) 650 W.

From the tensile test, we found that the fracture location is the weld zone for every sample; however, strength varies for every experiment. For Experiment 1, we obtained 898 MPa UTS and 767 MPa as yield strength, which is higher than of the base material, but elongation of the former is much lower than that of the latter. This sample can be considered to have brittle characteristics. The fracture morphology for the sample of experiment no. 1 is shown in Figure 4a, from which it is seen that cleavages and river patterns were created on the fracture surface. For Experiment 2, we found 811 MPa UTS and 660 MPa yield strength, which is lower than of the base material, and elongation is 43%, which is a reasonable value, which results in a longer existence and can withstand the same load for more time than the material of Experiment 1. So, this sample is not as brittle as the sample of Experiment 1, although we can say this welded sample is of ductile nature. The fracture morphology for the sample of Experiment 2 is shown in Figure 4b, from where it is seen that there were existences of few dimples and cleavage patterns with some micro voids in the fracture surface. For Experiment 3, we have 1007 MPa UTS, 603 MPa yield strength, and 62% elongation. Among them, UTS and elongation are both much higher than those of the base material and also compared to other welded samples, but yield strength is lower than in other experiments. This welded sample has the best longevity among all the experimental welded samples and can tolerate the load for the maximum time. This demonstrates that this welded sample has more ductility than that of the samples welded in the previous two experiments. The fracture morphology for the sample of Experiment 3 is shown in Figure 4c, which reveals that this welded sample accommodates apparent equiaxed dimple patterns and a small number of quasi-cleavages with few micro voids on the fracture surface. For Experiment 4, we obtain 692 MPa UTS, 520 MPa yield strength, and 9% elongation, which are very low compared to all other welded samples. This welded sample could not stand the load and broke rapidly. So, this sample is very much brittle in nature compared to all other welded samples. The fracture morphology for the sample of Experiment 4 is shown in Figure 4d, from which we can notice that, to a large extent, cleavage surfaces and river patterns were produced on the fracture surface. Previously, we noticed cleavages and river patterns on the fracture surface in Experiment 1, but in Experiment 4, more cleavage surface and river patterns were formed. A comparatively smoother face was formed at the fracture surface in Experiment 4, which was the reason for the brittle nature. In Experiment 1, the fracture surface was less smooth than in Experiment 4. In Experiment 2, the fracture surface contained a rough region, compared to Experiments 1 and 4. In Experiment 3, the fracture surface contains an extremely rough region, and this kind of morphology confirms that the fracture manners of this sample are more ductile in nature than the samples welded in other experiments.

### 3.3. Micro Hardness Test

**Figure 5:**
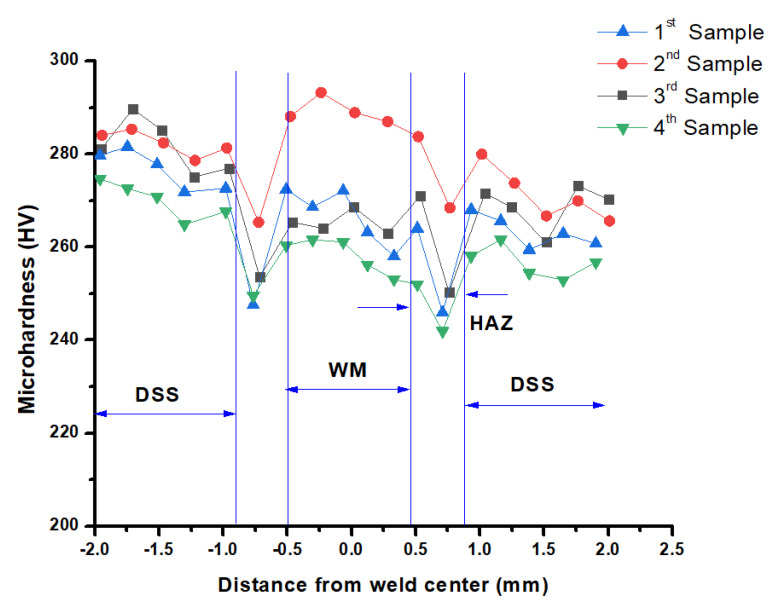
Micro hardness of different samples.

The Vickers microhardness profile of all welded samples through the base material, heat affected zone (HAZ), and weld zone were calculated and graphed in Figure 5. Microhardness values are in the range of 290–300 HV in welded regions, 270–285 HV in the heat affected zone (HAZ), and 250–265 HV in the base metal. It is evident, regarding these figures, that microhardness is decreasing incessantly from the fusion zone to the base material. The divergence of hardness between fusion and base zone is due to switching in metallurgical phase constituents [24]. Formations of greater quantities of intermetallic compounds over and above the development of bainite formation due to a higher cooling rate are also reasons for discrepancy among hardness. A higher cooling rate is capable of restraining the composition of softening in the fusion zone. Due to this higher cooling rate, we can have improved hardness in the fusion zone as an end result [30,31].

Hardness has a relation with grain size. From the Hall–Petch equation, σ_s_ = σ_0_ + kd^−1/2^, where σ_s_ is yield strength (MPa), σ_0_ is a constant, k is a constant, and d is grain size (mm); it can be said that, with reduced grain size, the yield strength will be elevated. The connection among hardness and yield strength can be explained with the help of the Tabor empirical formula, which is HV = C × σ_s_, where HV is hardness (Vickers scale), C is a constant in the range between 2.7 and 3.1, and σ_s_ is yield strength (MPa). Consequently, the correlation linking the hardness and grain size can be described as HV = C (σ_0_ + kd^−1/2^). Therefore, this equation implies that hardness will be enhanced with reductions in grain size, and vice versa. Different process parameters cause average heat input difference, which leads to increases and decreases in cooling rate, resulting in a dissimilarity of microhardness in fusion zone. Intended for every experiment, the weld zone provides elevated hardness in comparison to the base zone. In case of some welded samples, a quick rise or fall in hardness can be seen in between base and weld zone, which is referred to as the heat affected zone. Due to the small grain size in weld zone, an improved and higher micro hardness can be obtained. Due to recrystallization and grain growth, a coarser grain size is found at HAZ, but in spite of that, measured hardness is higher than in the base zone for some weld samples as a result of the carbide precipitates by the side of grain boundaries of the HAZ zone as well as of the weld zone [32].

## 4. Conclusions

This investigational research work intends to scrutinize the consequences of varying laser power, beam diameter, and welding speed on the excellence of butt weld along with base material by the means of tensile properties, fractography, and micro hardness. These subsequent denouements can be stated from the above research:

1. It is observed that ultimate tensile strength (UTS), elongation, and yield strength vary with different process parameters. We can conclude that, with the parameters used in Experiment 3, the correct amount of heat input is delivered to the sample, and the required amount of heat penetration was completed in the weld joints, since it gives maximum strength and elongation; therefore, laser power = 600 W, welding speed = 7 mm/s, and beam diameter = 0.7 mm are the optimum process parameters with which we obtain good quality butt joints. When process parameters increase, to some limit, elongation increases, but UTS shows a different nature. After exceeding that limit of process parameters, a drastic fall was shown in both the UTS and elongation properties.
2. A fracture location was found at the weld zone in every experimental sample. However, some of the welded samples were very brittle in nature. The samples with cleavage surface and river patterns at the fracture surface reveal a smooth area over that zone, which is more likely to be brittle. If the surface is rough and there is a minimum existence of cleavages and river patterns, then it is naturally more ductile. However, micro voids and dimples were also present in the fracture surfaces of welded samples.
3. The micro hardness of the weld zone is much higher than of the base zone for every welded sample. The discrepancy of hardness between fusion and base zone is due to toggling in metallurgical phase constituents. Developments of additional amounts of intermetallic compounds and growth of bainite formation as a result of higher cooling rate are also causes of discrepancies between hardness. A higher cooling rate can control the configuration of softening in the fusion zone. As a result of this higher cooling rate, we can achieve better hardness in the fusion zone. Micro hardness depends on grain size in an inverse manner. The weld zone consists of a smaller grain size than the base, resulting in greater hardness in the weld zone. For some welded samples, a sharp rise or fall was noticed in between weld and base zone, which is called the heat affected zone. Due to recrystallization and grain growth, larger sized grains have been formed at HAZ, but in spite of that, the hardness was higher than in the base zone for some weld samples due to carbide precipitates by the side of grain boundaries of the HAZ and weld zones.

## Figures and Tables

**Table 1:** Process parameters of laser welding.

Parameters	Values
Laser power (W)	500, 550, 600, 650
Wavelength (nm)	1064
Scanning speed (mm/s)	5, 6, 7, 8
Laser beam diameter (mm)	0.50, 0.60, 0.70, 1
Frequency (Hz)	20
Shielding gas (Argon) flow rate (liter/min)	6
Workpiece length (mm)	100
Workpiece width (mm)	20
Workpiece thickness (mm)	2

**Table 2:** Chemical composition and mechanical properties of the base material.

Grade	C	Cr	Ni	Mo	N	Mn	Cu	UTS (MPa)	Yield Strength (MPa)	Elongation (%)
S32520	0.03	24–26	5.5–8	3–4	0.2–0.35	1.5	0.5–2	862	745	36

**Table 3:** Experimental values of mechanical properties for the welded samples with different process parameters.

Exp No.	Power (W)	Welding Speed (mm/s)	Beam Diameter (mm)	UTS (MPa)	Elongation (%)	Yield Strength (MPa)
1	500	5	0.50	898	19 %	767
2	550	6	0.60	811	43 %	660
3	600	7	0.70	1007	62 %	603
4	650	8	1	692	9 %	520

## Data Availability

Not applicable.
